# Effects of collagen-derived bioactive peptides and natural antioxidant compounds on proliferation and matrix protein synthesis by cultured normal human dermal fibroblasts

**DOI:** 10.1038/s41598-018-28492-w

**Published:** 2018-07-11

**Authors:** Suzanne Edgar, Blake Hopley, Licia Genovese, Sara Sibilla, David Laight, Janis Shute

**Affiliations:** 1 Portsmouth, University of Portsmouth, Portsmouth, PO1 2DT, UK; 2 Minerva Research Labs, 1-6 Yarmouth Place, London, W1J 7BU UK

## Abstract

Nutraceuticals containing collagen peptides, vitamins, minerals and antioxidants are innovative functional food supplements that have been clinically shown to have positive effects on skin hydration and elasticity *in vivo*. In this study, we investigated the interactions between collagen peptides (0.3–8 kDa) and other constituents present in liquid collagen-based nutraceuticals on normal primary dermal fibroblast function in a novel, physiologically relevant, cell culture model crowded with macromolecular dextran sulphate. Collagen peptides significantly increased fibroblast elastin synthesis, while significantly inhibiting release of MMP-1 and MMP-3 and elastin degradation. The positive effects of the collagen peptides on these responses and on fibroblast proliferation were enhanced in the presence of the antioxidant constituents of the products. These data provide a scientific, cell-based, rationale for the positive effects of these collagen-based nutraceutical supplements on skin properties, suggesting that enhanced formation of stable dermal fibroblast-derived extracellular matrices may follow their oral consumption.

## Introduction

The biophysical properties of the skin are determined by the interactions between cells, cytokines and growth factors within a network of extracellular matrix (ECM) proteins^1^. The fibril-forming collagen type I is the predominant collagen in the skin where it accounts for 90% of the total and plays a major role in structural organisation, integrity and strength^2^. A complex network of interlaced collagen fibrils in the dermis provides support to the epidermis, and together with elastin and microfibrils gives the skin its elasticity and resilience^1^. In addition, proteoglycans and polymeric oligosaccharides, including abundant hyaluronic acid, play a key role in skin hydration.

Collagen I, elastin and proteoglycans, the three major groups of dermal ECM proteins, are secreted mainly by dermal fibroblasts activated by TGFβ, a multifunctional growth factor regulating the expression, deposition and turnover of skin extracellular matrix proteins^1^. Production of collagen and of the other components of the extracellular matrix is high when there is a sufficient level of mechanical tension on fibroblasts. When this tension is reduced, for example with age, the production of the matrix proteins falls and there is an increase of matrix-degrading enzymes^3^. The mature interstitial collagen fibrils are resistant to most proteolytic enzymes, but are susceptible to degradation by the collagenolytic matrix metalloproteinases MMP-1, MMP-8 and MMP-13^4^. Elastolytic MMPs include the macrophage metalloelastase MMP-12 and the weakly elastolytic MMP-3 which is expressed by fibroblasts.

The skin is subject to intrinsic (chronological) and extrinsic (environmental and lifestyle factors including UV radiation and smoking) ageing, which are both associated with histopathological and immunohistochemical changes^5^. Intrinsic ageing is characterised by cell senescence^6^, and altered levels of collagen^7^, elastin^8^ and glycosaminoglycans, including hyaluronic acid^9^. In extrinsic ageing, there is loss of reticular collagen and an accumulation of disorganised elastic fibres and glycosaminoglycans. Photo-aged skin (UV-irradiated) displays alterations of the extracellular matrix, with an increase in the expression of matrix metalloproteinases and collagenases^10^. Increased expression and activity of MMPs, notably MMP-1, MMP-3 and MMP-9^11,12^, has been associated with photo-ageing, and a direct effect of UV on the integrity of elastic microfibril associated proteins and on the elastin network has been suggested^8,13^.

A major cause of ageing-related skin damage is thought to be a consequence of decreased antioxidant defences leading to increased levels of intracellular reactive oxygen species (ROS). These form through aerobic metabolism and stimulate signal transduction resulting in the increased expression of MMPs and decreased collagen I synthesis^14^. The generation of ROS has been directly associated with protein damage, and with up-regulation in the expression and activity of MMPs in intrinsically and extrinsically aged skin^15,16^. Moreover, the ECM proteins can work as skin photo-sensitizers, enhancing the genotoxicity of a given dose of UV irradiation, contributing therefore to skin photo-ageing^17^. With age, the imbalance between synthesis and degradation of the ECM proteins leads to glycation and the consequent formation and accumulation of AGEs (advanced glycation end-products), which are a hallmark of age-related diseases^18^. Further, with age, the ability to replenish collagen naturally decreases by about 1% per year^19^. Thus, the administration of antioxidants might help in counteracting ROS-induced signs of ageing^20^. Along this line, it was shown that the administration of antioxidants can decrease oxidative stress in a model of prematurely ageing mice^21^. Moreover, clinical studies have shown that the oral administration of antioxidants can help improve skin condition in photo-aged skin^22,23^ and UV-induced erythema^24^.

In addition, evidence from placebo-controlled clinical studies supports the notion that daily oral consumption of collagen peptides derived by hydrolysis of native porcine and piscine collagen improves the density and integrity of the collagen network, hydration and elastic properties of normal skin^25,26^. Further, dietary supplements combining piscine collagen peptides with other active ingredients including hyaluronic acid, antioxidants, vitamins and minerals improve the appearance of the ageing skin^27–29^. However, the cellular mechanisms underpinning these observations remain to be elucidated.

The aim of this *in vitro* study is to investigate the effects on normal human dermal fibroblast synthesis of collagen I and elastin, release of transforming growth factor-β (TGF-β), plasminogen activator inhibitor-1 (PAI-1), matrix metalloproteinases (MMP), MMP-1 and MMP-3, and elastin degradation of collagen bioactive peptides, alone and in combination with other bioactive compounds found in two different collagen-based nutraceutical supplements previously reported to increase skin elasticity *in vivo*^28,29^.

Our hypothesis is that the antioxidant activity associated with the additives within these nutraceutical products interacts with the effects of the collagen peptides to enhance the stability of matrix proteins by inhibiting release of MMP-1 and MMP-3 in dermal fibroblast culture.

## Materials and Methods

### Test products

The collagen peptides, natural antioxidants and other bioactive molecules tested in this study are those found in the collagen-based nutraceutical supplements ACTIVE GOLD COLLAGEN^®^ (ACTIVE) and GOLD COLLAGEN^®^ FORTE (FORTE), which are manufactured by Minerva Research Labs (London, UK). The collagen peptides component (0.3–8 kDa; Peptan^®^ by Rousselot) was tested on normal primary human dermal fibroblasts in culture in the absence and presence of a full combination of other active ingredients at the concentrations shown in Table 1. These data are presented in the main results section. The collagen peptides were also tested following addition of individual active ingredients in the combinations shown in Table 1 to investigate possible additive effects of individual components. These data are described in the main results section and are presented graphically in Supplementary Figs S3–S8.

**Table 1:** The concentrations at which collagen peptides alone and in combination with other individual bioactive constituents of the nutraceuticals, ACTIVE and FORTE, were tested in normal dermal fibroblast cultures.

ACTIVE GOLD COLLAGEN^®^ constituents			Antioxidant activity	Combinations tested	
Code	Constituent	µg/ml	TE (µM)	No.	Addition
C	Hydrolysed piscine collagen	2000	76	1	C
HA	Hyaluronic acid	0.8	nd	2	C + HA
G	Glucosamine hydrochloride	400	nd	3	C + HA + G
CA	L-carnitine	400	10	4	C + HA + G + CA
M	Dried Maca root extract	8	93	5	C + HA + G + CA + M
P	Piperine (Piper Nigrum seed extract)	0.6	41	6	C + HA + G + CA + M + P
**GOLD COLLAGEN** ^®^ **FORTE constituents**					
**Code**	**Constituent**	**µg/ml**	**TE (µM)**	**No**.	**Addition**
C	Hydrolysed piscine collagen	2000	76	1	C
CS	Carnosine	24	44	2	C + CS
Q10	CoEnzyme Q10	10	19	3	C + CS + Q10
R	Resveratrol (red wine powder extract)	1	338	4	C + CS + Q10 + R
O	Borage seed oil/Primrose oil	10	95	5	C + CS + Q10 + R + O
HA	Hyaluronic acid	16	6	6	C + CS + Q10 + R + O + HA
P	Piperine (Piper Nigrum seed extract)	0.6	41	7	C + CS + Q10 + R + O + HA + P
L	Lycopene (tomato pulp extract)	0.04	64	8	C + CS + Q10 + R + O + HA + P + L
A	Acai berry extract	12	115	9	C + CS + Q10 + R + O + HA + P + L + A
PO	Pomegranate juice (concentrated)	8	95	10	C + CS + Q10 + R + O + HA + P + L + A + PO

The antioxidant activity measured as Trolox equivalents (TE) by peroxyl scavenging is shown for the individual constituents (mean, n = 4); nd = not detected. Combination number 6 for the ACTIVE constituents and combination number 10 for the FORTE constituents correspond to ‘All’.

### Collagen Peptides Production

Peptan^®^ collagen peptides are produced by hot water extraction of the endogenous collagen from fish skin, filtration, concentration, subsequent standardized and controlled enzymatic hydrolysis, sterilization and spray-drying. The production follows GMP guidelines and is HAACP-controlled in a IFS and ISO certified plant.

### Molecular Weight distribution

The molecular weight of Peptan^®^ collagen peptides is between 0.3 and 8 KDa. The molecular weight distribution of these collagen peptides is determined by high performance size exclusion chromatography (HPSEC) using an Agilent HPLC, 1260 Infinity series (G1316A, G1329B, G1311C, G1315D) with a TSKgel SWXL precolumn und a G2000SWXL column (Tosoh Bioscience). Analysis is performed with the WinGPC software (PSS). Samples are eluted from the column with 170 mM phosphate buffer containing 15% acetonitril and monitored with UV detection. Calibration is performed with the Narrow Calibration Standard (Low FILK).

### Amino Acids composition

The samples were hydrolysed in 9 M HCl at 110 C for 20 h, and subsequently derivatized and stabilized by 6-aminoquinolyl-N-hydroxysuccinimidyl carbamate (AccQ-Fluor reagent kit WAT052880). These fluorescent derivatives were separated by RP-HPLC on a Waters 2695 Alliance HPLC Separation Module and detected by a fluorescent detector. Quantification is performed by the Software Waters – Empower 3 using standards for each amino acid (Amino Acid Standard H WAT088122). Peptan^®^ collagen peptides are characterised by a high content of Glycine, Hydroxyproline/Proline and Glutamic Acid which represent 56% of the total amino acids. These collagen peptides comprise also Arginine (8%), Alanine (8%), essential amino acids (16%) and other amino acids (12%).

### Cell Culture

Normal adult human dermal fibroblasts (NHDF) were purchased from Lonza (Walkersville, MD, USA) and grown in fibroblast growth medium-2 (FGM), containing 2% FBS (Lonza), at 37 °C and 5% CO_2_. Cells *at passage 3–7* were seeded into 24 and 96 well plates at a density of 5 × 10^4^ cells/well, and 5 × 10^3^ cells/well for protein analysis and proliferation assays respectively. The fibroblasts were grown for 16 hours, quiesced for 24 h in medium containing 0.3% FBS and then treated for 48 hours with combinations of the bioactive constituents of ACTIVE or FORTE (Table 1) at a concentration found in a 1:50 dilution of the whole product. The rationale for testing components at this dilution is based on the absorption and distribution of avian collagen peptides of similar composition in an animal model, as detailed in the Supplementary Material.

These treatments were added in FGM containing 0.3% FBS, supplemented with 100 µM L-ascorbic acid and 100 µg/ml > 500 kDa dextran sulphate (Sigma-Aldrich, St. Louis, USA), which will be referred to hereafter as ‘crowded medium’^30,31^. Under these conditions cells remained viable, as confirmed by their adherence to cell culture plates and proliferation over 48 hours, as described in section 2.7. In all experiments, cell culture supernatants were collected, cleared by centrifugation at 1000 g for 10 minutes (min) at 4 °C and stored at −80 °C until assayed.

### Collagen I

Cells were washed twice with phosphate buffered saline. Cell-associated collagen was solubilised by the addition of 0.5 M acetic acid (250 µl/well) and gentle agitation at 4 °C for 32 hours, before adding 0.1 mg/ml pepsin from porcine mucosa (Sigma-Aldrich) and continuing agitation for a further 16 hours. Pepsin digestion was then inhibited with 2 µg/ml pepstatin A (Sigma-Aldrich). Cell samples were stored at -80 °C until assayed. A novel quantitative immuno-blot assay for native collagen I was developed and validated by Western blot analysis of TGFβ-activated NHDF cells and supernatants (Supplementary Fig. S1). Standards (0.625–10 ng calf skin collagen I, Sigma Aldrich) and samples (100 µl) diluted with PBS (acid-soluble cell fraction, 1:10, and supernatants, 1:20), were loaded onto nitrocellulose membrane using a vacuum manifold to create protein dots. Blots were dried, blocked with 5% dried skimmed milk powder and 2% Tween 20 in PBS and incubated overnight at 4 °C with rabbit anti collagen I antibody (Abcam, Cambridge, UK) at 200 ng/ml in block buffer. The blots were then incubated with 50 ng/ml goat anti rabbit-HRP antibody (Dako, Glostrup, Denmark) for 1 hour at room temperature. Blots were visualised using chemiluminescence (Thermo-Fisher, Waltham, USA).

### Elastin

Cells were lifted with trypsin-EDTA (Sigma-Aldrich) and diluted 3:1 with 1 M oxalic acid to 0.25 M oxalic acid. Cell samples were incubated at 95–100 °C for 2 hours, with intermittent mixing, and the entire cell and supernatant samples from each well were assayed separately for solubilised elastin using the Fastin elastin assay kit (Biocolor, Carrickfergus, UK), following manufacturer’s instructions.

### AP-1 analysis

NHDF were lysed in 20 mM TRIS-HCl, pH 7.6, containing 150 mM NaCl plus 1% Triton X-100, lysis buffer and 2 × protease inhibitors, protease cocktail I (Calbiochem), and Complete® protease inhibitor (Roche), PhosSTOP® phosphatase inhibitor (Roche), 4 mM MgCl_2_ and benzonase (Sigma-Aldrich) at 1:1000 dilution in sample buffer (35 ul per well of 24-well plate).

Samples were mixed with sample buffer and separated on 10% SDS-PAGE and proteins transferred to nitrocellulose using semi-dry electrophoresis. Membranes were blocked with 3% dried skimmed milk powder in TRIS (20 mM) buffered saline (150 mM) plus 0.1% Tween-20, and stained with rabbit anti-p-c-jun (Ser 63/73) at 200 ng/ml, and secondary goat-anti-rabbit-HRP (Sigma-Aldrich) at 1:2000 dilution. Chemiluminescence (Promega) was used to detect bands following 30 mins exposure in the ChemiDoc imager (BioRad).

### ELISAs

Cells were lysed using hypotonic lysis buffer (1% Triton X-100 in 10 mM Tris-HCl buffer, pH 7.4) containing 2 × protease cocktail I inhibitors (Merck (Calbiochem), Darmstadt, Germany) and stored at −80 °C until assayed. Supernatants were analysed neat for TGF-β, PAI-1 and desmosine, and at a 1:25 dilution for MMPs -1 and-3. Total TGF-β, total MMP-1, total MMP-3, TIMP-1 and PAI-1 ELISA kits from R&D systems (Abingdon, UK) were used according to the manufacturer’s instructions.

A desmosine ELISA was performed using an in-house competitive ELISA as previously described^32^. In brief, the wells of a 96-well Nunc maxisorp microtitre plate were coated with 250 ng desmosine-egg-albumin complex (Elastin Products Company (EPC), Missouri, USA) in 100 µl bicarbonate buffer, pH 9.6 (Sigma-Aldrich), overnight at 4 °C. Desmosine standards (EPC) were prepared in the range 0–2000 ng/ml in 100 mM Tris-HCl pH 7.2, containing 0.1%Tween-20. Standards and samples (100 µl) were added to 200 µl of a 1:3000 rabbit anti-desmosine serum (EPC) and incubated at 37 °C for 30 min. Plates were washed, and standards and samples (100 µl in duplicate) were added to the wells and incubated for 2 h at 4 °C. Biotinylated swine anti-rabbit (Dako) antibody, 100 µl at 1:1000 dilution, was added to each well and incubated for 1 h at room temperature. Streptavidin-HRP complexes (Vector Labs), 100 µl at 1:1000 dilution, were added for 30 min at room temperature, followed by substrate (5.5 mM o-phenylene-diamine solution in TRIS-citrate buffer pH 6). Reactions were stopped by the addition of 100 ul 2 M H_2_SO_4_ and the plate read at 490 nm.

### Cell Proliferation

The proliferation assay was performed after 48 hours of cell culture, as described in section 2.2, using the CyQuant NF assay kit (Thermo Fisher) according to the manufacturer’s instructions. A standard curve to calculate cell number was prepared with 100–50,000 cells per well, in triplicate, which were allowed to adhere for 4 h, then stained with CyQuant dye binding solution for 40 min, and fluorescence was measured at excitation/emission wavelengths 485/530.

### Antioxidant activity

The antioxidant activity of the collagen peptides and the other bioactive ingredients tested was measured using a pyranine-based procedure to evaluate the total peroxyl scavenging capacity^33,34^. The method is based on the ability of antioxidants to prevent the bleaching of pyranine (200 μM) by peroxyl radicals generated from AAPH (2,2′-azo-bis- (2-amidinopropane) hydrochloride (200 mM). Trolox, a water-soluble analogue of vitamin E was used as a standard and values were expressed as Trolox equivalents (TE). Samples (25 μl) were mixed with 25 μl pyranine and incubated for 3 min at 37 °C. AAPH (50 μl) was added and the reaction monitored at 454 nm every minute for 80 min. The lag phase to bleaching was determined for samples and Trolox standards in the range 0 to 0.5 mM. Reagents were all from Sigma-Aldrich.

### Statistical analysis

Fibroblast responses are presented as mean ± sem and were analysed by one-way ANOVA followed by Fisher’s LSD post-hoc test using GraphPad Prism, version 7, software. Correlation between the cumulative antioxidant activity of individual bioactives and elastin, MMP-1 and MMP-3 levels in NHDF cultures was analysed by one-tailed Pearson correlation coefficient test. Differences where p < 0.05 were considered to be statistically significant.

## Results

**Figure 1:**
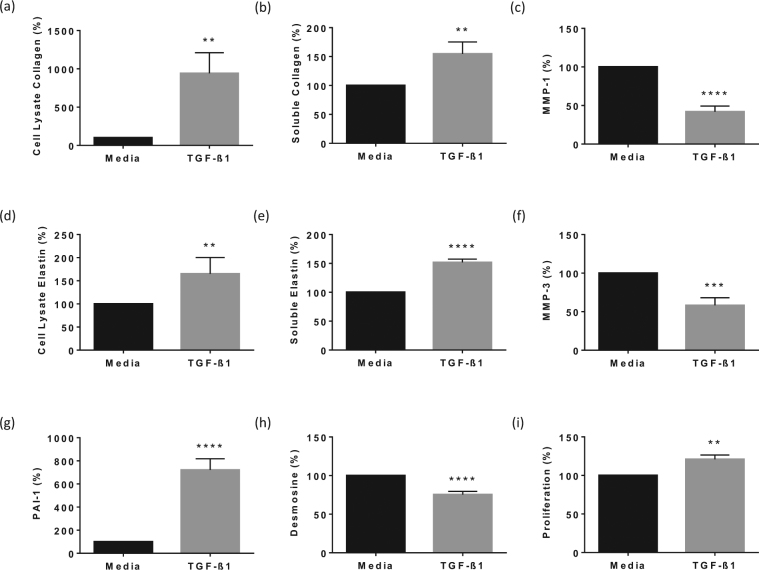
The effect of TGF-β1 on protein levels in normal human dermal fibroblasts (NHDF) cultures. Proteins were measured in cell lysates and/or supernatants of NHDF grown in 24 well plates and activated with TGF-β1 (5 ng/ml) for 48 hours. Data was expressed as % of media control which was normalised to 100% in each experiment. Collagen I and elastin were measured in cell lysates (**a,d**) and supernatants (**b,e**), MMP-1 (**c**), MMP-3 (**f**), PAI-1 (**g**) and desmosine (**h**) were measured in supernatants. Proliferation was measured using the CyQuant assay (**i**). The data is presented as mean ± SEM. **indicates P < 0.01, ***P < 0.001, ****P < 0.0001. Media control values in (**a**) = 0.6 ± 0.2 ng/well, n = 11 (**b**) = 2.5 ± 0.6 ng/well, n = 11 (**c**) = 22.7 ± 3.5 ng/ml, n = 7 (**d**) = 11.4 ± 1.9 µg/well, n = 7 (**e**) = 6.5 ± 0.8 µg/well, n = 7 (**f**) = 23.7 ± 6.4 ng/ml, n = 6 (**g**) = 1618.7 ± 126.3 pg/ml, n = 8 (**h**) = 1655 ± 109.2 ng/ml, n = 8 (**i**) = 25564.5 ± 5758.9 fluorescence units, n = 4.

It has previously been reported that TGF-β at 5 ng/ml strongly stimulates collagen synthesis by embryonic pulmonary fibroblasts in crowded culture^30^. Therefore, because TGF-β controls expression, deposition and turnover of collagens and other extracellular matrix proteins in the skin^1^, and primary dermal fibroblast responses to TGF-β as a positive control in cultures crowded with high molecular weight dextran sulphate have not previously been reported, we initially tested the effect of TGF-β (5 ng/ml) on NHDF protein synthesis and proliferation after 48 hour incubation under similarly crowded conditions, compared to media alone (Fig. 1). The values for baseline concentrations of all proteins in quiescent cultures are presented in the legend to Fig. 1. Significantly more collagen I was found in the supernatant (2.5 ± 0.6 ng/well) compared to the cell layer (0.6 ± 0.2 ng/well). These values were normalised to 100% to test the relative effect of TGF-β. TGF-β stimulated a significant increase in collagen I in the cell lysate (942.0 ± 268.3%; Fig. 1a) and supernatant (154.6 ± 20.7%; Fig. 1b) compared to media alone (100%). Unlike collagen I, significantly more elastin was found, in quiescent cultures, associated with cell layers (11.4 ± 1.9 µg/well) compared to supernatants (6.5 ± 0.8 µg/well). Further, TGF-β significantly increased elastin in the cell lysate (165.1 ± 35.1%; Fig. 1d) and supernatant (151.8 ± 5.5%; Fig. 1e). TGF-β significantly decreased total MMP-1 (42 ± 7.4%; Fig. 1c), total MMP-3 (58.1 ± 9.9%; Fig. 1f) and desmosine (75.4 ± 4.1%; Fig. 1h) in the culture supernatants, significantly increased PAI-1 (721.2 ± 96.5%; Fig. 1g) in the supernatant and significantly increased the proliferation of NHDF (121.0 ± 5.4%; Fig. 1i) compared to the effect of media alone, normalised to 100%.

The effects of the addition of collagen peptides alone (C) and in combination with all the other bioactive and antioxidant constituents (All) present within the two nutritional supplements, ACTIVE and FORTE, at the concentrations described in Table 1, were tested and compared to the effect of media alone (designated as the 100% value in all experiments). In parallel, the effect of adding individual constituents to the collagen peptides, in the combinations described in Table 1, were tested and these results are presented in Supplementary Figs S3–S8. Collagen I, elastin and TGF-β synthesis by NHDF was measured in both the cell lysate and supernatant. PAI-1, MMP-1, MMP-3, TIMP-1 and desmosine were measured in the supernatant alone where they were most abundant.

**Figure 2:**
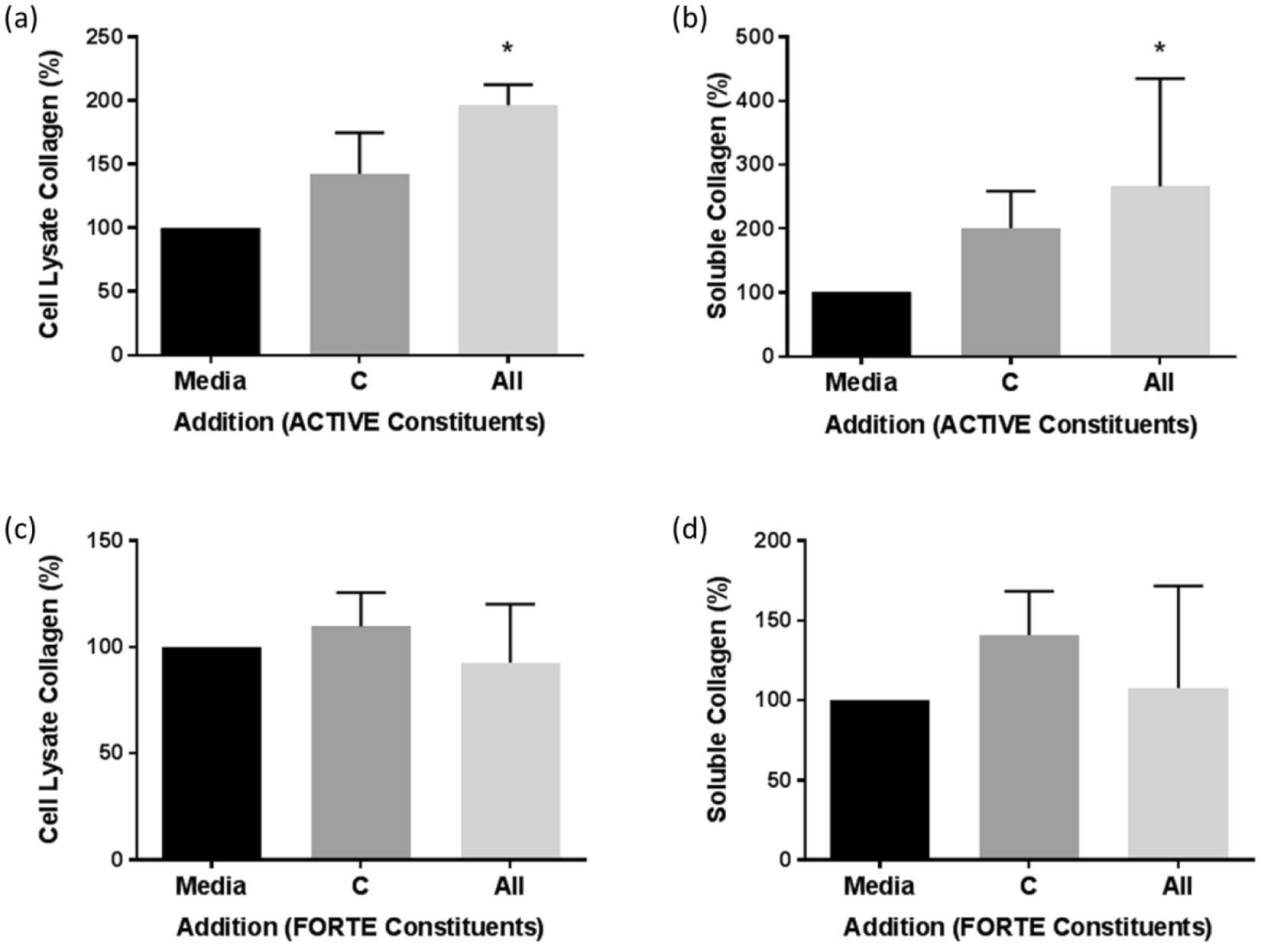
The effect of collagen peptides alone and in combination with all other bioactives on collagen I levels in NHDF cultures. Full length native collagen I was measured in cell lysates (**a,c**) and supernatants (**b,d**) of NHDF grown in 24 well plates and incubated in media plus collagen peptides alone (C) and in combination with the other constituents listed in Table 1 (All) of ACTIVE (**a,b**) or FORTE (**c,d**) for 48 hours. Data was expressed as % of media control, normalised to 100% in each experiment and is presented as mean ± SEM of 3 independent experiments. *Indicates P < 0.05. Media control in (**a**) = 0.7 ± 0.3 ng/well, (**b**) = 1.3 ± 0.4 ng/well, (**c**) = 0.4 ± 0.1 ng/well and (**d**) = 2.9 ± 0.9 ng/well.

Collagen I was found predominantly in soluble form when cells were grown in media alone (Fig. 2, legend). Although collagen peptides alone had no significant effect on collagen I in the supernatant, soluble collagen I was significantly increased in response to the combination of collagen peptides with the other five ACTIVE constituents tested (All; 265.8 ± 169% of media control; Fig. 2b). Similarly, although cell-associated collagen in the cell lysate was not significantly increased in response to collagen peptides alone (C; 142.5 ± 32.2%), in combination with all other ACTIVE constituents cell lysate collagen was significantly increased (All; 196.2 ± 16.2%; Fig. 2a). Considering the individual combinations of ACTIVE constituents (Table 1, Fig. Supplementary Fig. S3a) all those including glucosamine in combination with hyaluronic acid and collagen peptides, but not hyaluronic acid or collagen peptides alone, significantly increased cell-associated collagen I (Supplementary Fig. S3a,c). In contrast, the combination of collagen peptides with the other nine constituents of FORTE (All) did not increase cell-associated or soluble collagen I (Fig. 2c,d).

**Figure 3:**
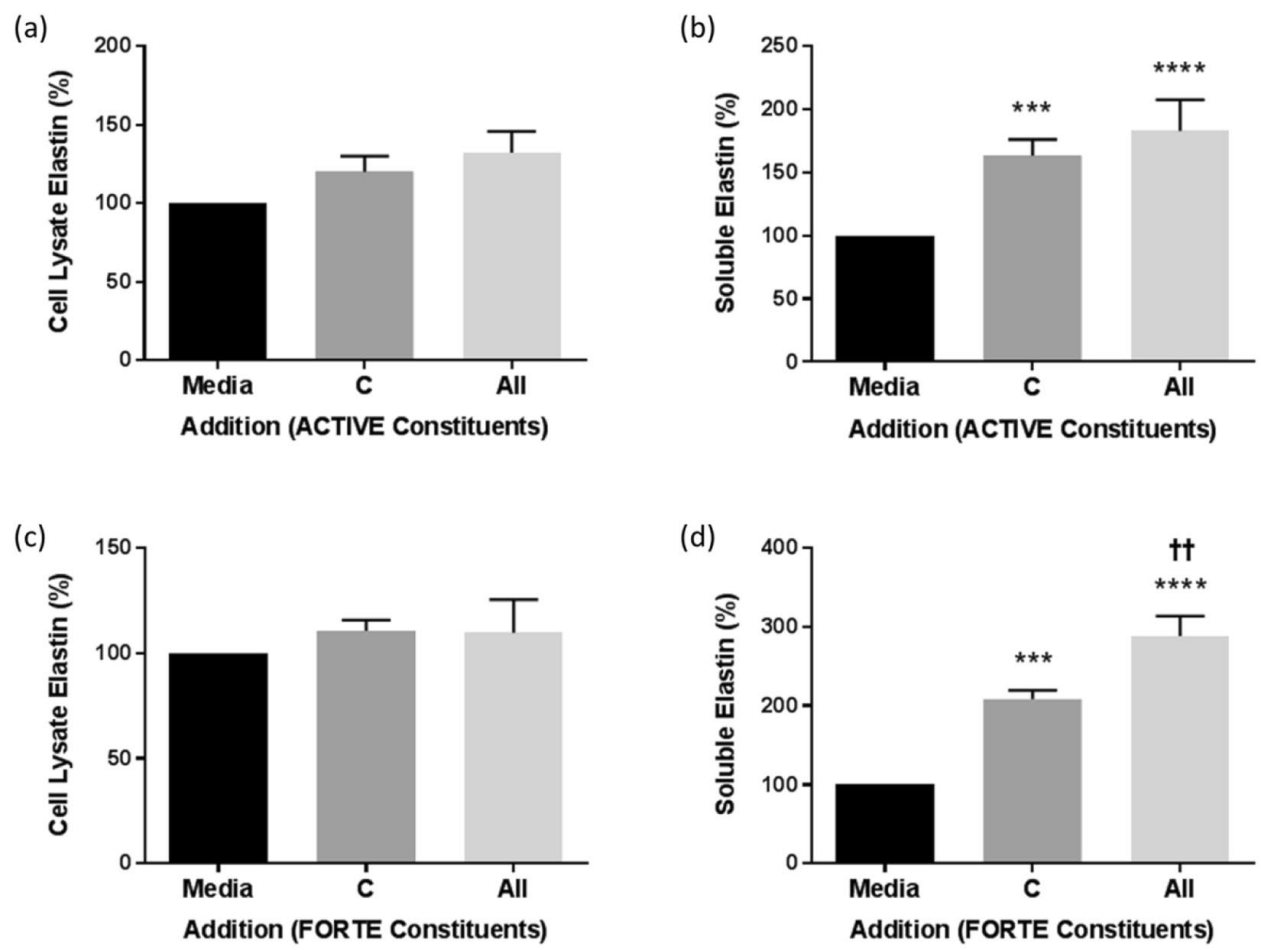
The effect of collagen peptides alone and in combination with all other bioactives on elastin levels in NHDF cultures. Elastin was measured in cell lysates (**a,c**) and supernatants (**b,d**) of NHDF grown in 24 well plates and incubated in media plus collagen peptides alone (C) and in combination with the other constituents listed in Table 1 (All) of ACTIVE (**a,b**) or FORTE (**c,d**) for 48 hours. Data was expressed as % of media control, normalised to 100% in each experiment and is presented as mean ± SEM of 4 independent experiments. **/†† indicates P < 0.01, ***P < 0.001, ****P < 0.0001. Media control in (**a**) = 15.2 ± 2.7 µg/well, (**b**) = 8.3 ± 1.7 µg/well, (**c**) = 10.8 ± 3.4 µg/well and (d) = 6.1 ± 1.7 µg/well. *Indicates groups compared to 0 (media alone), ^†^indicates groups compared to 1 (collagen peptides).

Unlike collagen, elastin was found predominantly in cell lysates in unstimulated cultures (Fig. 3, legend). The addition of collagen peptides alone (C) significantly increased the amount of soluble elastin (Fig. 3b,d). Moreover, in combination with the nine other bioactive constituents of FORTE (All) the effect was further significantly enhanced (Fig. 3d).

Although all combinations (Table 1, Supplementary Fig. S4c,d) of the FORTE and ACTIVE constituents increased soluble elastin in the supernatant compared to media alone, only FORTE constituents stimulated a further significant increase in soluble elastin compared to the collagen peptides alone (Supplementary Fig. S4d). However, there was no significant increase in the cell-associated elastin in cell lysates on addition of collagen peptides to any combination with other bioactives (Fig. 3a,c, Supplementary Fig. S4a,b).

Addition to dermal fibroblast cultures of the whole ACTIVE and FORTE products (including acidity regulators, stabilisers, natural sweeteners, flavourings and vitamins) at 1:50 dilution, to match the tested concentrations of individual components, significantly increased soluble elastin to 173 ± 27.9% and 187 ± 40%, respectively, of the media control value.

**Figure 4:**
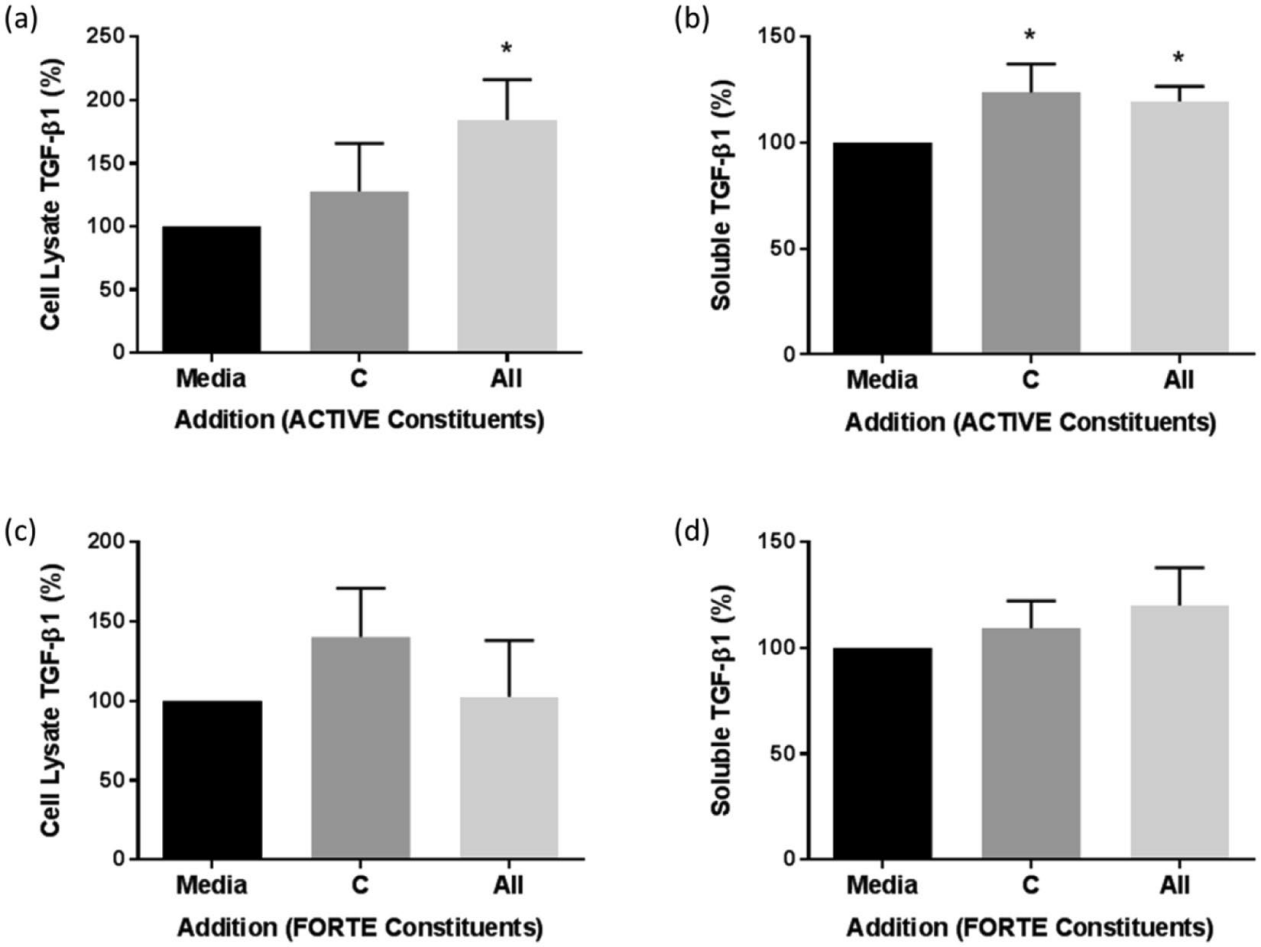
The effect of collagen peptides alone and in combination with all other bioactives on TGF-β1 levels in NHDF cultures. TGF-β1 was measured in cell lysates (**a,c**) and supernatants (**b,d**) of NHDF grown in 24 well plates and incubated in media plus collagen peptides alone (C) and in combination with the other constituents listed in Table 1 (All) of ACTIVE (**a,b**) or FORTE (**c,d**) for 48 hours. Data was expressed as % of media control, normalised to 100% in each experiment and is presented as mean ± SEM of 3 independent experiments for ACTIVE and 3 independent experiments for FORTE. *Indicates P < 0.05. Media control in (**a**) = 29.72 ± 12.2 pg/ml, (**b**) = 49.1 ± 10.3 pg/ml, (**c**) = 3.6 ± 1.2 pg/ml and (**d**) = 25 ± 7.5 pg/ml.

We first considered whether the observed increases in collagen and elastin concentrations were due to an autocrine effect of TGF-β released by dermal fibroblasts under specific culture conditions. However, TGF-β was detected at low (pg/ml) levels (Fig. 4, legend) and found in an inactive form (i.e. requiring acid activation for detection). TGF-β was significantly increased in cell lysates (All; 184.2 ± 32%; Fig. 4a) and in supernatants (All; 119.6 ± 7%; Fig. 4b) in response to collagen peptides in combination with all ACTIVE constituents. However, there was no significant increase in TGF-β in either the supernatant (Fig. 4d) or cell lysate (Fig. 4c) in response to addition of the constituents of FORTE.

Active TGF-β is a potent inducer of PAI-1 in NHDF culture supernatants (Fig. 1g). However, PAI-1 was not increased under any culture condition (data not shown), confirming that TGF-β was present only at low levels and in an inactive form. Further, while TGF-β strongly induced AP-1 activation, detected as an increase in phospho-c-jun in cell lysates (Supplementary Fig. S2) the collagen peptides and other bioactives under investigation did not (data not shown).

**Figure 5:**
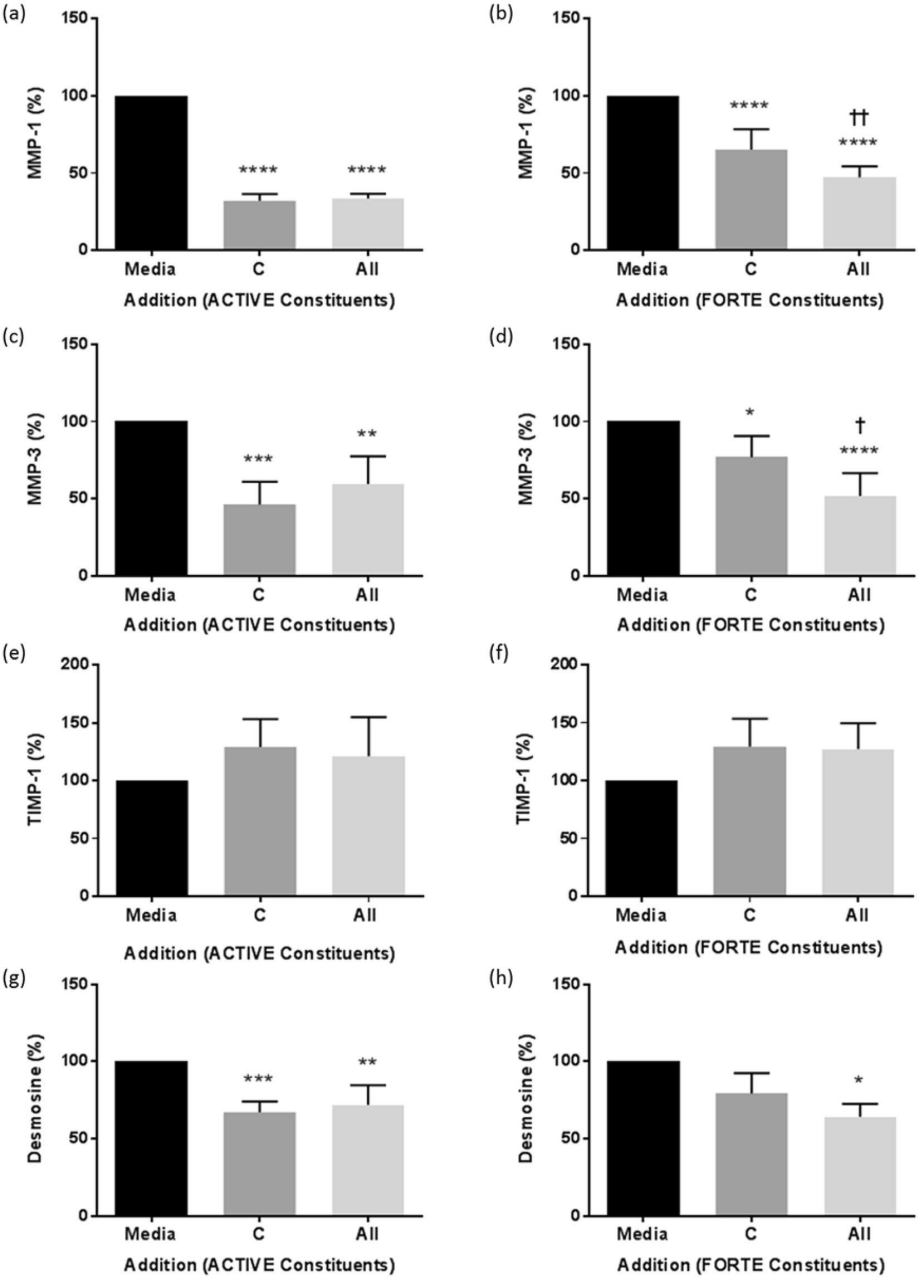
The effect of collagen peptides alone and in combination with all other bioactives on MMP-1, MMP-3 and TIMP-1 levels and elastin breakdown in NHDF cultures. MMP-1, MMP-3, TIMP-1 and desmosine were measured in supernatants of NHDF grown in 24 well plates and incubated in media plus collagen peptides alone (C) and in combination with the other constituents listed in Table 1 (All) of ACTIVE (**a,c,e,g**) or FORTE (**b,d,f,h**) for 48 hours. Data was expressed as % of media control, normalised to 100% in each experiment and is presented as mean ± SEM of 3 independent experiments. *^/†^Indicates P < 0.05, **^/††^P < 0.01, ***P < 0.001, ****P < 0.0001. Media control in (**a**) = 26.4 ± 8.2 ng/ml, (**b**) = 5.6 ± 1.7 ng/ml, (**c**) = 25.5 ± 8.6 ng/ml, (**d**) = 0.6 ± 0.3 ng/ml, (**e**) and (**f**) = 314.1 ± 34.6 ng/ml, (**g**) = 1.6 ± 0.5 µg/ml and (**h**) = 1.7 ± 0.5 µg/ml. *Indicates groups compared to 0 (media alone), ^†^indicates groups compared to 1 (collagen peptides).

Because the measured increases in collagen and elastin potentially reflect reduced degradation by MMP-1 and MMP-3, respectively, we measured levels of these proteases and their cognate inhibitor, TIMP-1, in culture supernatants. A highly significant decrease in MMP-1 protein levels in supernatants (Fig. 5a,b) was seen in response to collagen peptides alone and the combination of all six ACTIVE constituents (All; 33.8 ± 3% of media control; Fig. 5a) and all ten FORTE constituents (All; 47.4 ± 7% of media control; Fig. 5b). The effect of the collagen peptides was further significantly increased on addition of the other constituents of FORTE (Fig. 5b). In addition, all combinations of ACTIVE and FORTE constituents significantly decreased MMP-1 levels in the supernatants (Supplementary Fig. S6a,b), but an additive effect of the FORTE constituents stimulated further significant decrease in MMP-1 protein levels compared to collagen peptides alone (Supplementary Fig. S6b).

Similarly, MMP-3 (Fig. 5c,d) was significantly decreased in supernatants in response to collagen peptides alone and in the presence of the six ACTIVE ingredients (All; 59.6 ± 18% of media control) and ten FORTE ingredients (All; 52 ± 14.8% of media control). The antioxidants and bioactive constituents of FORTE further significantly enhanced the effect of the collagen peptides (Fig. 5d). In fact, collagen peptides and all combinations of ACTIVE and FORTE constituents significantly reduced MMP-3 levels in supernatants (Supplementary Fig. S6c,d), but an additive effect of the FORTE constituents stimulated further significant decrease in MMP-3 protein levels compared to collagen peptides alone (Supplementary Fig. S6d).

In parallel with the significant decrease in MMP-1 and MMP-3 expression, a small but non-significant increase in TIMP-1 levels were observed (Fig. 5e,f) with collagen peptides alone that was not altered by addition of other constituents of ACTIVE or FORTE supplements.

In parallel with the decrease in MMP-3 concentrations, desmosine as a marker of elastin breakdown (Fig. 5g,h) was significantly decreased in response to collagen peptides in combination with all six ACTIVE constituents (All; 72 ± 12.6%; Fig. 5g), and all ten FORTE constituents (All; 64.3 ± 8.2%; Fig. 5h). All combinations (Supplementary Fig. S7a,b) of ACTIVE constituents, but only some combinations of FORTE constituents caused a significant decrease in the breakdown of elastin.

**Figure 6:**
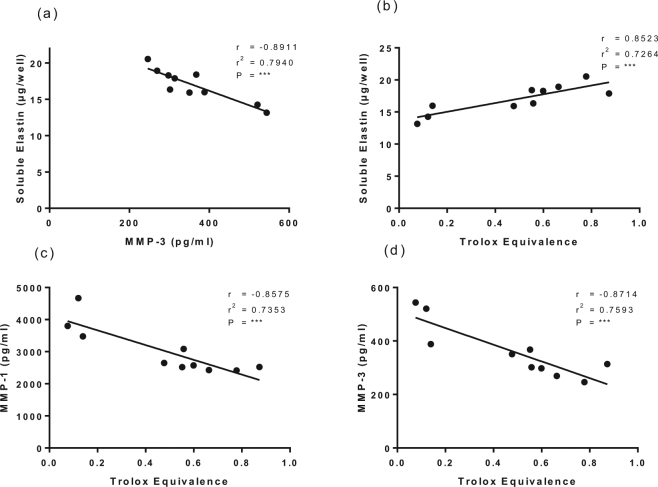
Correlation between MMP-3 and soluble elastin **(a)** and the cumulative antioxidant activity of individual bioactives in FORTE and soluble elastin (**b**), MMP-1 (**c**) and MMP-3 (**d**) levels in NHDF cultures. A one-tailed Pearson correlation coefficient test was used to analyse significance of the data. **Indicates P < 0.01, ***P < 0.001, ns = no significance.

In view of the lack of evidence for autocrine effects of endogenous TGF-β in the cultures, we considered whether the observed inhibition of MMP-1 and MMP-3 expression was related to the total antioxidant activity of the added bioactives. In particular, the individual constituents of FORTE appeared to have an additive effect (Supplementary Fig. S6), and when added all together they significantly enhanced the effect of the collagen peptides (Fig. 5,b,d and h). The antioxidant activity of the collagen peptides and each of the bioactives under investigation was measured as peroxyl scavenging activity, and expressed as Trolox equivalents (Table 1). The data in Fig. 6 show the strong, significant, negative correlations between the cumulative antioxidant activity of the added FORTE constituents and MMP-1 and MMP-3 expression levels (Fig. 6c,d). Further, there is a significant negative correlation of soluble elastin with MMP-3 activity (Fig. 6a) and a significant positive correlation with antioxidant activity (Fig. 6b), indicating that basal expression and release of MMPs is driven by reactive oxygen species and that antioxidant activity added to the cultures was responsible for the further significant inhibition of MMP-3 release (Fig. 5d) and increase in soluble elastin (Fig. 3d, Supplementary Fig. S6d) beyond that seen with the collagen peptides alone.

**Figure 7:**
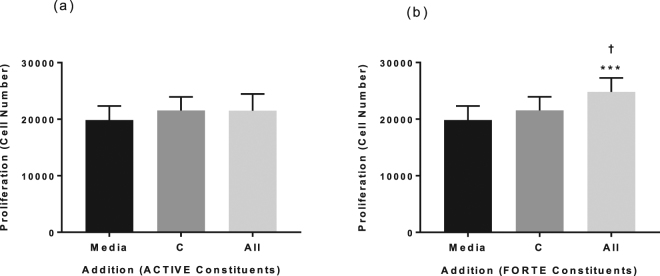
The effect of collagen peptides alone and in combination with other constituents of ACTIVE and FORTE on proliferation of NHDF. Proliferation was measured in NHDF grown in 96 well plates and incubated in media plus collagen peptides alone (C) and in combination with the other constituents listed in Table 1 (All) of ACTIVE (**a**) or FORTE (**b**) for 48 hours. Data was expressed is presented as mean ± SEM of 4 independent experiments. Media control value is 19,848 ± 2,476 cells. ^†^Indicates P < 0.05, ***P < 0.001. *Indicates groups compared to 0 (media alone), ^†^indicates groups compared to 1 (collagen peptides).

Although there was no significant effect on proliferation (Fig. 7) when adding the collagen peptides alone, the combination of all ten constituents of FORTE significantly stimulated NHDF proliferation over 48 hours (All; 123.8 ± 6.5%; Fig. 7b). In addition, incubation of NHDF with HA, a constituent of ACTIVE (Supplementary Fig. S8a), and carnosine, a constituent of FORTE (Supplementary Fig. S8b) resulted in significantly increased cell proliferation that was not further enhanced by the addition of other bioactives.

## Discussion

TGF-β is a well-recognised pro-fibrotic growth factor that promotes the deposition of ECM proteins, whilst limiting their degradation. The pro-fibrotic effects of TGF-β were confirmed in our model in which normal human dermal fibroblasts were cultured under conditions of macromolecular crowding to mimic the extracellular matrix environment of the skin. Exogenously added TGF-β1 significantly increased fibroblast proliferation, collagen and elastin synthesis and release of PAI-1, while inhibiting release and MMP-1 and MMP-3 and the breakdown of elastin. These effects are mediated by SMAD and AP-1 dependent signalling pathways that stimulate synthesis of collagen-I via Smad3 and PAI-1 via AP-1^35^ and interact to repress MMP-1 and MMP-3 gene expression^36,37^. Redox signalling also plays an important role in the pro-fibrotic effects of TGF-β^38^.

Media crowded with macromolecules such as high molecular weight dextran sulphate (0.01% w/v) was reported to increase collagen synthesis by embryonic pulmonary fibroblasts 20–30 fold, compared to normal media^31^ and soluble procollagen was completely processed into insoluble collagen by dermal fibroblasts^39^, although the collagen was deposited as aggregates and not fibrils^30^. Using a sensitive in-house immunoblot method to detect collagen I, the presence of soluble collagen I indicated processing was incomplete in our study. However, it is possible that the high assay sensitivity detected more soluble collagen I than previous studies.

Collagen peptides significantly increased cell-associated collagen, but only in the presence of glucosamine (Supplementary Fig. S3). Positive effects on collagen levels may be associated with the ability of glucosamine to stabilise collagen matrices by reducing MMP synthesis, as demonstrated in synovial fibroblasts^40^. Although we found no peroxyl scavenging activity associated with glucosamine (data not shown), superoxide/hydroxyl-radical scavenging antioxidant activity of glucosamine hydrochloride has previously been reported, and it was suggested that glucosamine hydrochloride could be effectively employed as an ingredient in functional food, to alleviate oxidative stress^41^. However, the lack of any significant effect of adding antioxidants such as resveratrol, CoQ10, acai berry, lycopene and pomegranate on collagen expression, despite significant inhibition of MMP-1 expression, indicates an effect of glucosamine on collagen synthesis that is not mediated through antioxidant activity.

In contrast, soluble elastin was significantly increased by collagen peptides alone and with all combinations of antioxidants and other bioactives tested. Each of the constituent antioxidants found in FORTE appeared to have an additive effect on total soluble elastin synthesis. Moreover, when added to fibroblast cultures as the whole product (diluted 1:50 to give the same concentrations of the tested individual components), both collagen-based nutraceuticals significantly (p < 0.05) increased soluble elastin almost two-fold. This increase in elastin expression reflects what has already been observed *in vivo*. A significant increase in skin firmness (Young’s elasticity) was demonstrated *in vivo* after 90 days of supplementation with both whole collagen-based supplements investigated in this study^28,29^. Low molecular weight avian collagen peptides of similar composition, when orally administered in rats, were shown to accumulate preferentially in the skin^42^, at concentrations which could be scaled to the concentration of peptides consumed daily in the test products (100 mg/ml) and used in our in vitro study (2 mg/ml). Thus our *in vitro* observations support those from our previous *in vivo* studies, validating the model we have used to investigate dermal fibroblast responses to individual components of the products.

The very low levels of TGF-β released (25–50 pg/ml) by dermal fibroblasts in this model, and the lack of effect of any of the additives on the levels of active TGF-β, indicated that an autocrine effect of TGF-β was unlikely, and this is evidenced by the lack of effect on AP-1 activation and PAI-1 expression.

Collagen peptides alone and in combination with antioxidants and other bioactives significantly reduced MMP-1 and MMP-3 expression, in the absence of any change in TIMP-1 levels. Notably, the constituents of FORTE appeared to have an additive effect on inhibition of MMPs expression, as was also seen for the increase in soluble elastin in culture supernatants. A major cause of ageing-related skin damage is thought to be due to increased levels of ROS and oxidative stress^14^ which directly and indirectly, through increased expression of MMPs, damage structural proteins^43^. MMPs -1, -2, -3 and -9 are mainly responsible for ECM damage and degradation, and can fully degrade collagen together. However, the only MMP that can damage intact collagen I fibres is MMP-1, while MMP-3 is capable of degrading elastin^43^. Protective effects of the collagen peptides and other constituents on elastin integrity are indicated by a decrease in desmosine, a cross-linked amino acid and specific marker of elastin breakdown, in the cultures. The negative correlation of soluble elastin concentration with MMP-3 supports the notion that MMP-3 is responsible for elastin breakdown and the generation of desmosine in the cultures.

The significant positive association between total peroxyl scavenging activity of the additives and soluble elastin content in the cultures may now suggest a mechanistic role for antioxidant activity in the observed increase in soluble elastin. Potentially the effect is mediated by increased elastin transcription in the presence of the antioxidants, as previously shown for the effect of the antioxidant N-acetylcysteine^44^ or decreased expression of MMP-1 and MMP-3, which was previously reported for the effect of the antioxidant Tiron^45^ and mitochondria-targeted vitamin E^46^ on dermal fibroblasts. The significant negative correlation of the MMPs with antioxidant activity indicates that the antioxidants are protecting the cells from constitutive intracellular ROS, activation of AP-1 signalling and MMP expression^14^. These effects transcend any potential activities associated with the physiological (100 µM) concentration of ascorbic acid added to the culture medium as a co-factor for prolyl and lysyl hydroxylases involved in collagen synthesis, since 88–98% of ascorbate disappears from culture medium within 24 h^47^ and we found the peroxyl scavenging activity of 100 µM ascorbic acid was low (6 µM TE).

Finally, collagen peptides in combination with antioxidants and other bioactives under investigation, but not alone, stimulated fibroblast proliferation, and the magnitude of the effect was similar to that seen with TGF-β. A strong effect of HA on dermal fibroblast proliferation was not further increased by other additions. While HA has not been shown to be directly mitogenic^48^, through facilitating proliferation in response to other mitogenic factors such as TGF-β^49^ and, in this instance, collagen peptides, HA may have an important but indirect role in cell proliferation. Similarly, the stimulating effect due to addition of carnosine on fibroblast proliferation was not further enhanced by the other additions. McFarland and Holliday (1999) showed in their classical experiments that carnosine enhances the proliferative potential of fibroblasts by protecting the cells from telomere shortening^50^. Together with stimulating effects on dermal fibroblast proliferation^51^ carnosine may play an important role in skin regeneration.

In conclusion, 48 hour incubation of dermal fibroblasts with collagen-derived peptides and other nutraceutical constituents increases structural ECM protein synthesis, particularly elastin, and decreases synthesis of MMPs -1 and -3 and the elastin degradation product desmosine. The effect of these collagen-based supplements and their constituents therefore support normal adult human dermal fibroblast function in terms of potentially increasing matrix stability in the skin. This effect is not likely to be mediated through TGF-β signalling pathways but is more likely correlated with antioxidant effects on dermal fibroblast MMP expression.

## Electronic supplementary material

Supplementary material and figures
