# Supplementary material for: Effects of collagen-derived bioactive peptides and natural antioxidant compounds on proliferation and matrix protein synthesis by cultured normal human dermal fibroblasts
Source: Sci Rep. 2018 Jul 11;8:10474. doi: 10.1038/s41598-018-28492-w (PMC6041269; doi:10.1038/s41598-018-28492-w)
Supplement: Supplementary file 1 — Supplementary material and figures [file 41598_2018_28492_MOESM1_ESM.docx]

**Effects of collagen-derived bioactive peptides and natural antioxidant compounds on proliferation and matrix protein synthesis by cultured normal human dermal fibroblasts**

**Short title: Effects of collagen and antioxidants on fibroblasts proliferation and protein synthesis**

**Suzanne Edgar^a^, Blake Hopley^a^, Licia Genovese^b^, Sara Sibilla^b*^, David Laight^a^, Janis Shute^a^.**

**^a^Institute of Biomedical and Biomolecular Sciences, University of Portsmouth, PO1 2DT, UK and ^b^Minerva Research Labs, 1-6 Yarmouth Place, London, W1J 7BU, UK.**

***Corresponding author:** Sara Sibilla, Research and Development Department, MINERVA Research Labs, 1-6 Yarmouth Place, London, W1J 7BU, UK; email; [ssibilla@minervalabs.com](mailto:ssibilla@minervalabs.com).

This work was performed in United Kingdom.

Number of words (not including not including Abstract, References and Figure Legends) = 4825

Number of references = 51

Number of Tables = 1

Number of Figures = 7

Number of Supplementary Figures = 8

**Supplementary Material**

Dilution of additives.

The collagen peptides, natural antioxidants and other bioactive molecules tested in this study are those found in the collagen-based nutraceutical supplements ACTIVE GOLD COLLAGEN® (ACTIVE) and GOLD COLLAGEN® FORTE (FORTE), which are manufactured by Minerva Research Labs (London, UK). These products contain 5 g peptides derived from hydrolysed piscine collagen in a total volume of 50 ml, which is for daily oral consumption.

The 1:50 dilution of the test products and constituents was based on the reported distribution of orally administered avian-derived collagen peptides (60 kBq, 3.6 x 10^6^ dpm) to the skin, 20,000 dpm/g tissue (Watanabe-Kamiyama M., 2010 et al.) in 5-week old Wistar rats (~150 g) and the report that skin is 19% of rat body weight (Brown, Delp, Lindstedt, Rhomberg & Beliles, 1997). Thus, of orally administered collagen peptides, 16% (570,000 dpm/28.5g tissue) was distributed in the rat skin.

The weight of the human adult dermis is 1848 g – 2500 g in reference woman and man, respectively (ICRP, 2002), which allows us to estimate 16% of 5 g collagen peptides as a single daily dose, distributed in 1848 g or 2500 g dermis tissue, (0.43 mg/g dermis in reference woman to 0.32 mg/g dermis in reference man). The final concentration of collagen peptides in our dermal fibroblast cultures was 2 mg/ml, to take into account the stable accumulation of collagen peptides in the skin compared to other organs (Watanabe-Kamiyama M., 2010 et al.) when consumed orally on a daily basis. The product, containing 100 mg/ml collagen peptides, was therefore diluted in the cell cultures by a factor of 1:50, and the same dilution factor was applied to the other ingredients found in the whole food supplement.

**Supplementary Figure S1.** A novel, highly sensitive immuno-dot-blot method to quantify collagen I.

Samples were quantified via the use of immuno-dot-blots, using a corresponding standard curve of calf skin collagen I, shown in (a), and then scanning the image into Quantiscan densitometry software (b). SDS-PAGE with Western blotting was used to confirm the integrity and identity of collagen I in the samples against a collagen I standard. (c) Lane 1= 1.5 µg collagen I calf skin standard from a gel analysing a range of collagen I concentrations to determine the sensitivity of the method. The two collagen I α- chain bands (α1(I)= 139 kDa, α2(I)= 129 kDa) are labelled with an arrow. Corresponding bands can be seen in NHLF samples, analysed on a separate gel, in lanes 2-7. Lanes 2, 4 and 6= NHDF cell lysates, lanes 3, 5 and 7= NHDF media supernatants. Lanes 2 and 3= untreated cell cultures, lanes 4 and 5= cultures treated with 5ng/ml TGF-β, lanes 6 and 7= cultures treated with 10 ng/ml TGF-β, for 48 hours.

0 ng

0.625 ng

1.25 ng

2.5 ng

5 ng

10 ng


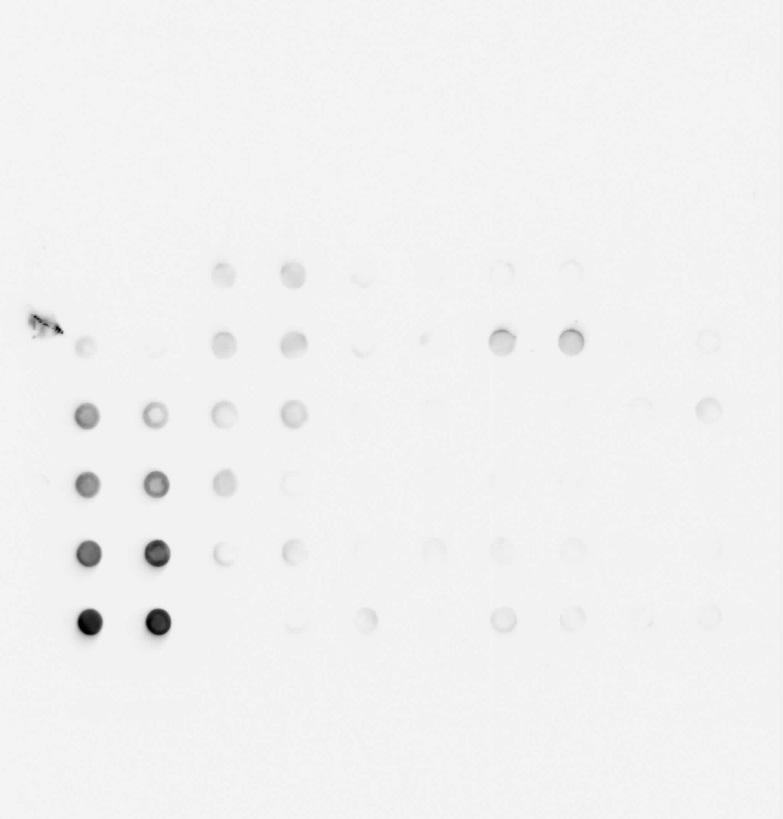

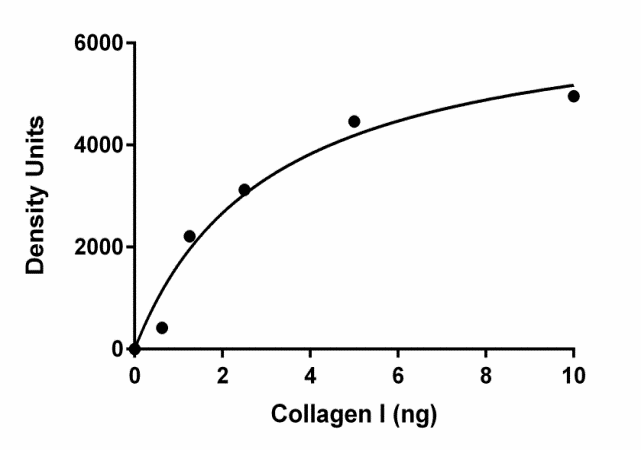


(a)

(b)

(c)

1

2

3

4

5

6

7


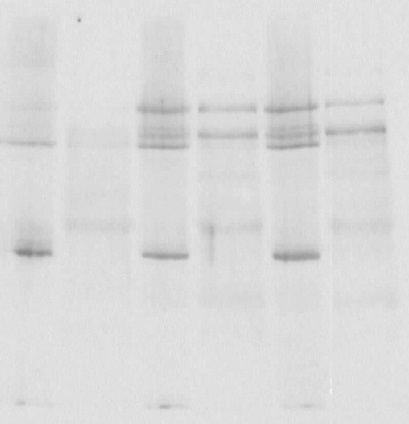

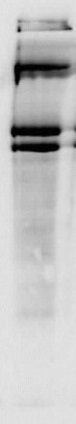


α1(I)

α2(I)

Supplementary Figure S1.

**Supplementary Figure S2.** Western blot analysis for AP-1 activation following TGFβ (5 ng/ml) stimulation of NHDF in crowded cultures and analysis of cell lysates. Activation of AP-1 was detected by staining for phospho-c-jun using an antibody (Santa Cruz p-c-Jun Antibody (Ser 63/73): sc-16312R). TGFβ strongly activated c-jun phosphorylation as indicated by the 45 kDa band. The high molecular weight band may reflect nuclear p-c-jun complexes.


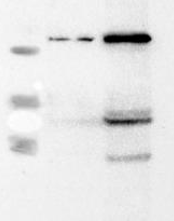


MW Media +TGFβ

100

60

40

Supplementary Figure S2.

**
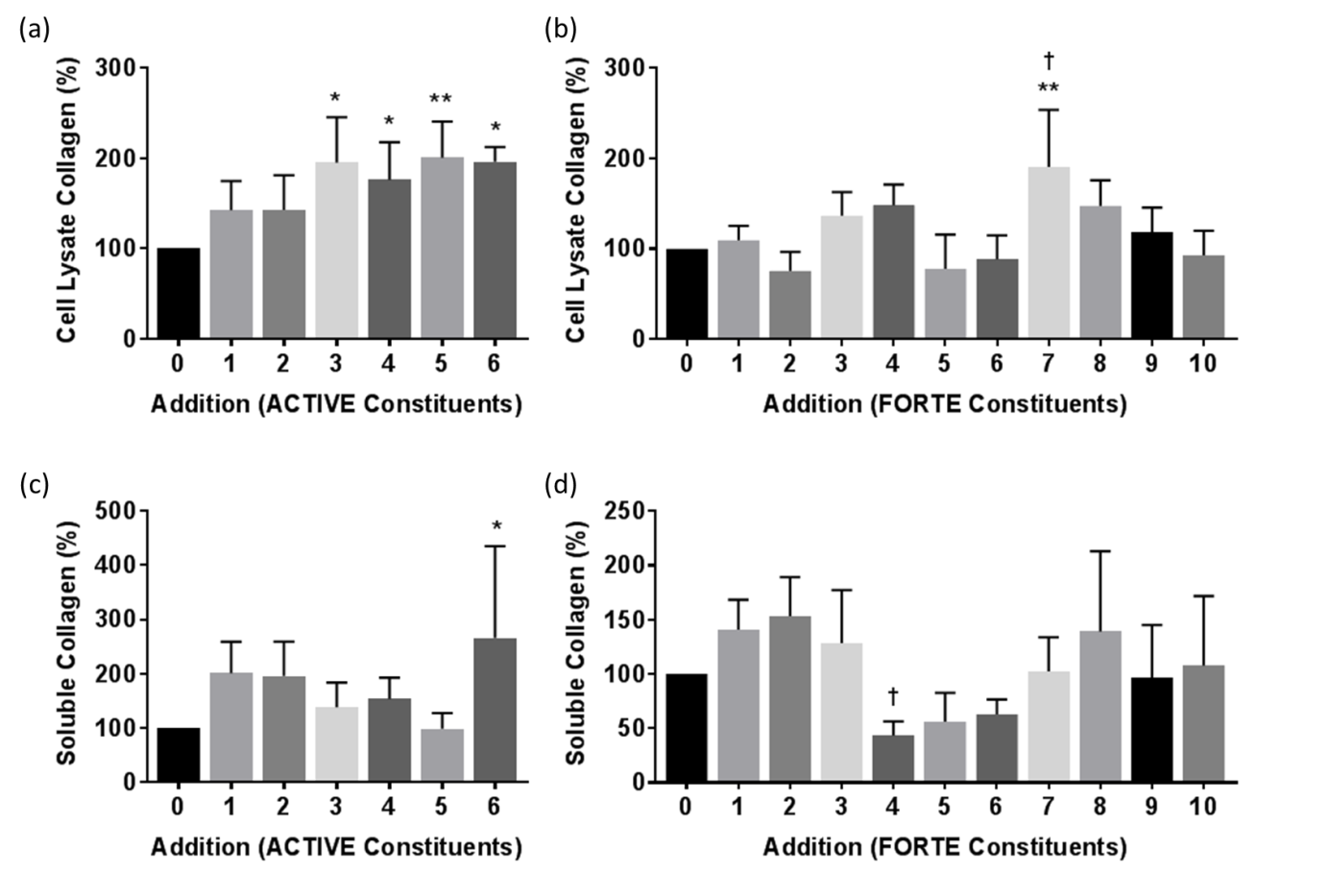
Supplementary Figure S3**. The effect of collagen peptides alone and following sequential addition of individual bioactives on collagen I synthesis by NHDF. Full length native collagen I was measured in cell lysates (a, b) and supernatants (c, d) of NHDF grown in 24 well plates and incubated in media alone (0), in the presence of collagen peptides (1) and following the addition of individual constituents of ACTIVE (a, c) or FORTE (b, d), as described in Table 1, for 48 hours. Data was expressed as % of media control, normalised to 100% in each experiment and is presented as mean ± SEM of 3 independent experiments. */† indicates P<0.05, ** P<0.01. Media control in (a)= 0.7 ± 0.3 ng/well, (b)= 1.3 ± 0.4 ng/well, (c)=0.4 ± 0.1 ng/well and (d)=2.9 ± 0.9 ng/well. * indicates groups compared to 0 (media alone), † indicates groups compared to 1 (collagen peptides).

Supplementary Figure S3.

**Supplementary Figure S4.** The effect of collagen peptides alone and following sequential addition of individual bioactives on elastin synthesis by NHDF. Elastin was measured in cell lysates (a, b) and supernatants (c, d) of NHDF grown in 24 well plates and incubated in media alone (0), in the presence of collagen peptides (1) and following the addition of individual constituents of ACTIVE (a, c) or FORTE (b, d), as described in Table 1, for 48 hours. Data was expressed as % of media control, normalised to 100% in each experiment and is presented as mean ± SEM of 4 independent experiments. † indicates P<0.05, †† P<0.01, *** P<0.001, **** P<0.0001. Media control in (a)=15.2 ± 2.7 µg/well, (b)=8.3 ± 1.7 µg/well, (c)=10.8 ± 3.4 µg/well and (d)=6.1 ± 1.7 µg/well. * indicates groups compared to 0 (media alone), † indicates groups compared to 1 (collagen peptides).

Supplementary Figure S4.
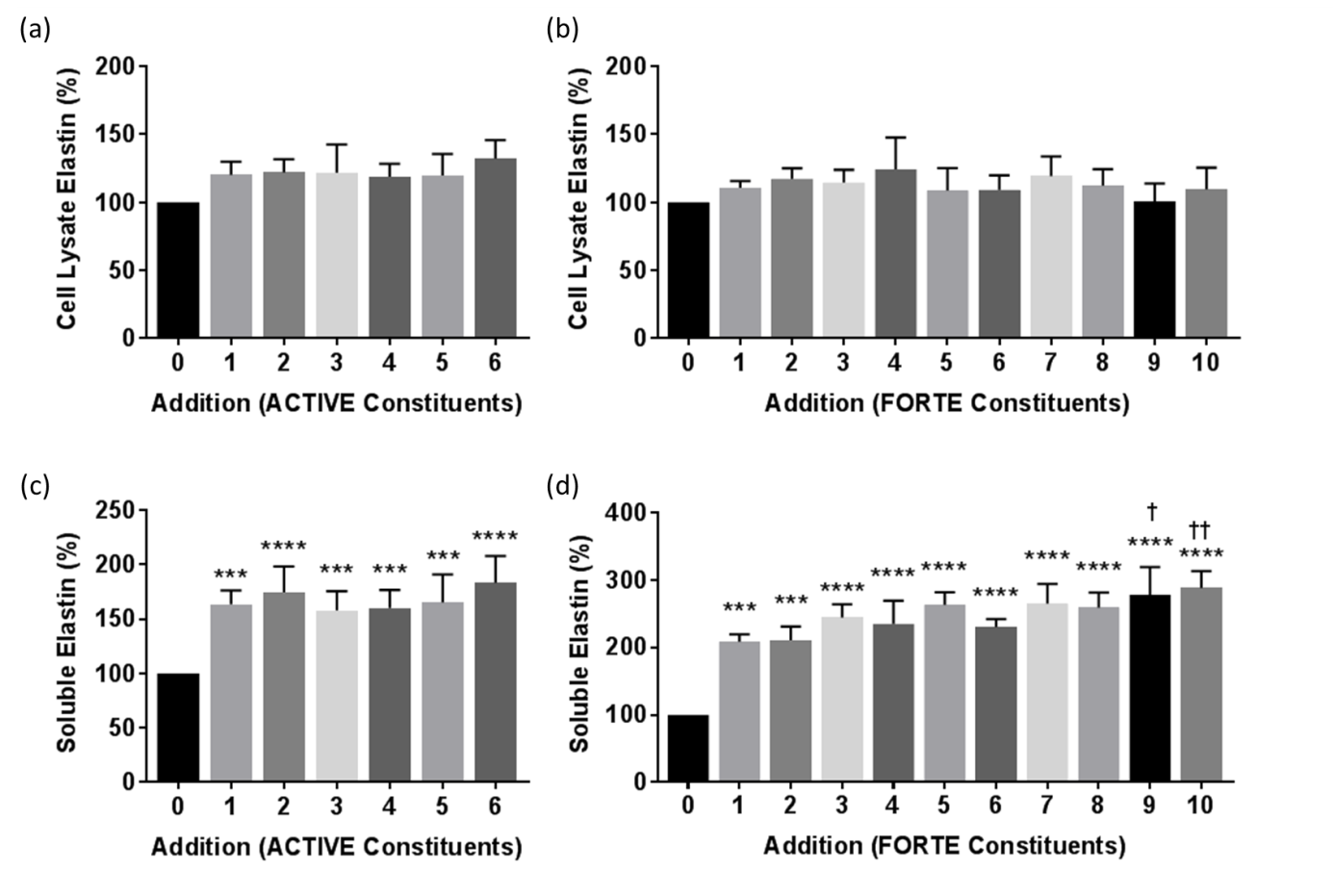


**
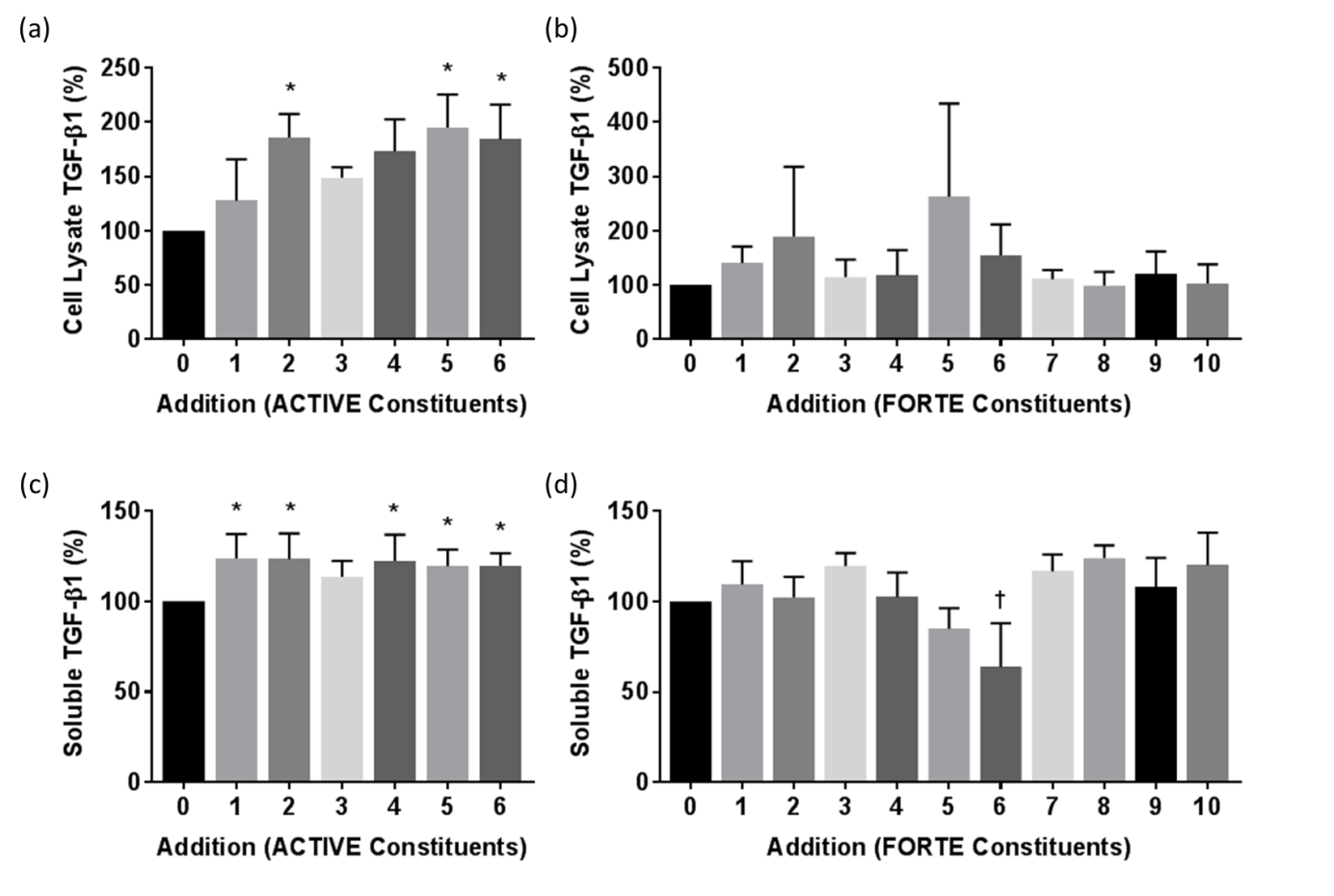
Supplementary Figure S5**. The effect of collagen peptides alone and following sequential addition of individual bioactives on TGF-β1 synthesis by NHDF. TGF-β1 was measured in cell lysates (a, b) and supernatants (c, d) of NHDF grown in 24 well plates and incubated in media alone (0), in the presence of collagen peptides (1) and following the addition of individual constituents of ACTIVE (a, c) or FORTE (b, d), as described in Table 1, for 48 hours. Data was expressed as % of media control, normalised to 100% in each experiment and is presented as mean ± SEM of 3 independent experiments for ACTIVE and 3 independent experiments for FORTE. */† indicates P<0.05. Media control in (a)=29.72 ± 12.2 pg/ml, (b)=49.1 ± 10.3 pg/ml, (c)=3.6 ± 1.2 pg/ml and (d)=25 ± 7.5 pg/ml. * indicates groups compared to 0 (media alone), † indicates groups compared to 1 (collagen peptides).

Supplementary Figure S5.

**
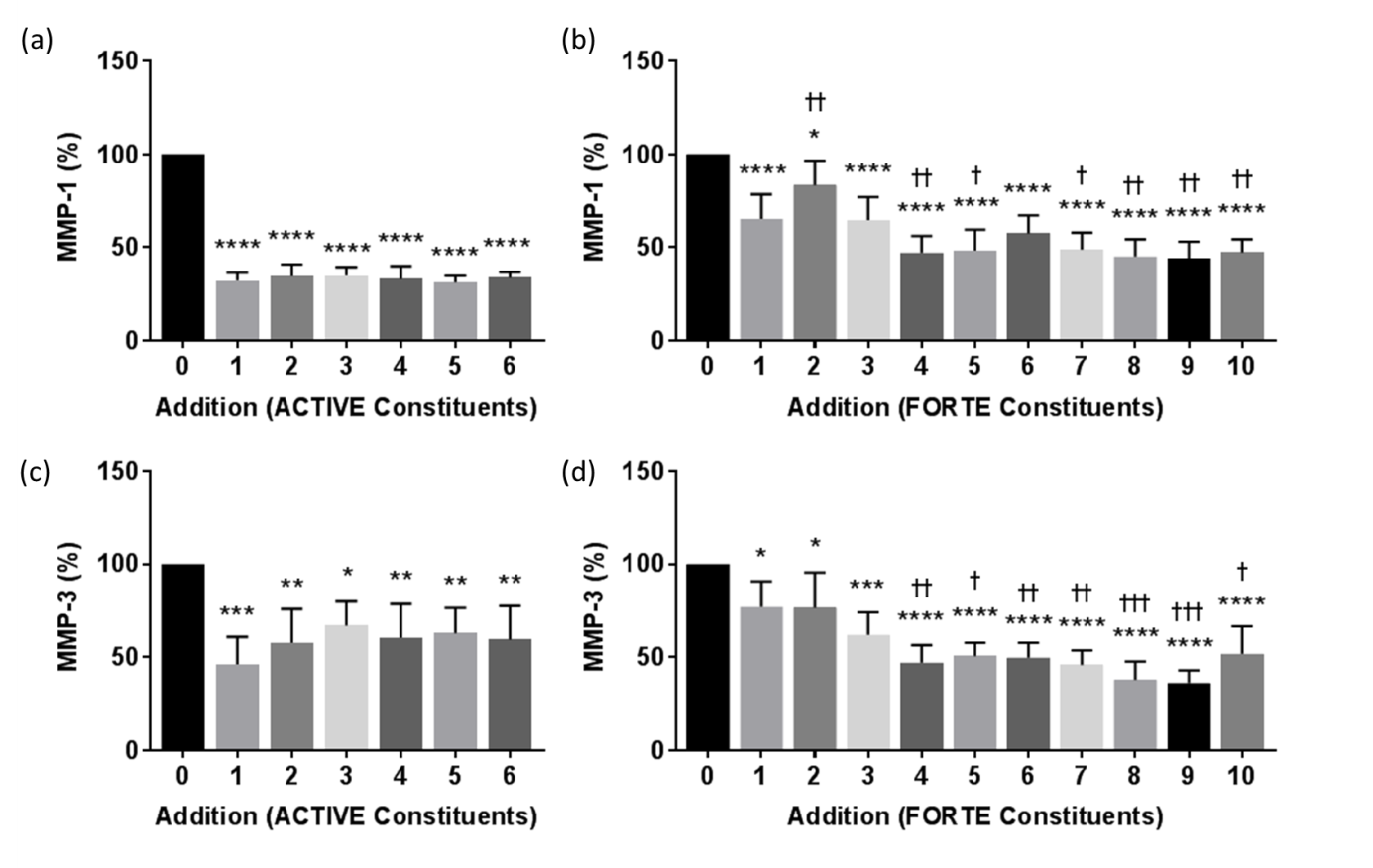
Supplementary Figure S6**. The effect of collagen peptides alone and following sequential addition of individual bioactives on MMP-1 and MMP-3 expression by NHDF. MMP-1 and MMP-3 were measured in supernatants of NHDF grown in 24 well plates and incubated in media alone (0), in the presence of collagen peptides (1) and following the addition of individual constituents of ACTIVE (a, c) or FORTE (b, d), as described in Table 1, for 48 hours. Data was expressed as % of media control, normalised to 100% in each experiment and is presented as mean ± SEM of 3 independent experiments. */† indicates P<0.05, **/†† P<0.01, ***/††† P<0.001, **** P<0.0001. Media control in (a)=26.4 ± 8.2 ng/ml, (b)=5.6 ± 1.7 ng/ml, (c)= 25.5 ± 8.6 ng/ml, (d)= 0.6 ± 0.3 ng/ml, (e) and (f)=314.1 ± 34.6 ng/ml. . * indicates groups compared to 0 (media alone), † indicates groups compared to 1 (collagen peptides).

Supplementary Figure S6.

**
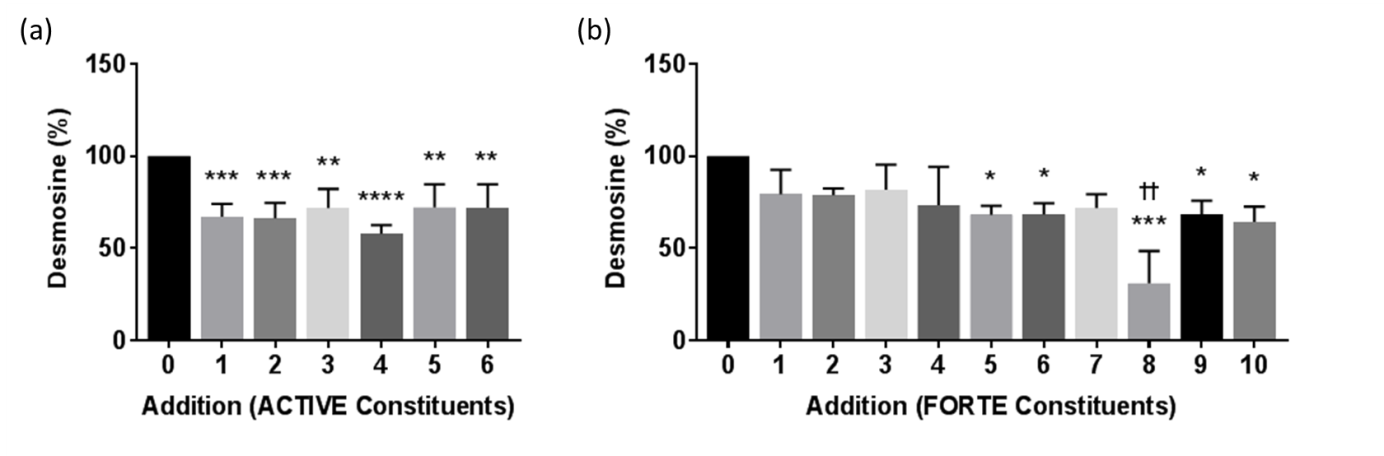
Supplementary Figure S7**. The effect of collagen peptides alone and following sequential addition of individual bioactives on desmosine synthesis by NHDF. Desmosine was measured in supernatants of NHDF grown in 24 well plates and incubated in media alone (0), in the presence of collagen peptides (1) and following the addition of individual constituents of ACTIVE (a) or FORTE (b), as described in Table 1, for 48 hours. Data was expressed as % of media control, normalised to 100% in each experiment and is presented as mean ± SEM of 3 independent experiments. * indicates P<0.05, **/†† P<0.01, *** P<0.001, **** P<0.0001. Media control in (a)=1.6 ± 0.5 µg/ml and (b)=1.7 ± 0.5 µg/ml. * indicates groups compared to 0 (media alone), † indicates groups compared to 1 (collagen peptides).

Supplementary Figure S7.

**Supplementary Figure S8**. The effect of collagen peptides alone and following sequential addition of individual bioactives on proliferation of NHDF. Proliferation was measured in NHDF grown in 96 well plates and incubated in media alone (0), in the presence of collagen peptides (1) and following the addition of individual constituents of ACTIVE (a) or FORTE (b), as described in Table 1, for 48 hours. Data was expressed as cell number, mean ± SEM of 4 independent experiments. Media control value is 19,848±2,476 cells. */† indicates P<0.05, **/†† P<0.01, *** P<0.001. * indicates groups compared to 0 (media alone), † indicates groups compared to 1 (collagen peptides).

(b)

(a)

Supplementary Figure S8.

**REFERENCES**

Brown RP, Delp MD, Lindstedt SL, Rhomberg LR, Beliles RP. Physiological parameter values for physiologically based pharmacokinetic models. Tox Industr Health 1997; 13: 407-416.

ICRP. Basic Anatomical and Physiological Data for Use in Radiological Protection Reference Values. Ann ICRP. 2002;32:5-526.

Watanbe-Kamiyama M, Shimizu M, Kamiyama S, Tagushi Y, Sone H, Morimatsu F, Shirakawa H, Furukawa Y, Komai M. Absorption and effectiveness of orally administered low molecular weight collagen hydrolysate in rats. J Agric Food Chem 2010; 58: 835-841.
